# Association of postoperative delirium with hypotension in critically ill patients after cardiac surgery: a prospective observational study

**DOI:** 10.1186/s13019-024-02958-7

**Published:** 2024-08-01

**Authors:** Saleh Mohammed Alhaj Othman, Mohammed Ali Ali Aziz, Gaber Musaed Ali Al-Mushiki, Chanyanud Sriwayyapram, Tecleab okubai, Gamil Al-Muwaffaq, Qin Xu, Mohammed Alqudaimi

**Affiliations:** Nanjing Medical University, Longmian Avenue No.101, Jiangning District, Nanjing, Jiangsu China

**Keywords:** Delirium, Postoperative hypotension, Surgery-related complications, Adult population, Open heart surgery, Postoperative care, Critical illness

## Abstract

**Background:**

Postoperative delirium (POD), an acute and variable disturbance in cognitive function, is an intricate and elusive phenomenon that occurs after cardiac surgery. Despite progress in surgical techniques and perioperative management, POD remains a formidable challenge, imposing a significant burden on patients, caregivers, and healthcare systems.

**Methods:**

This prospective observational study involved 307 patients who underwent cardiac surgery. Data on the occurrence of delirium, clinical parameters, and postoperative characteristics were collected. A multivariate analysis was performed to assess the relationship between POH and POD.

**Results:**

Sixty-one patients (21%) developed delirium, with an average onset of approximately 5 days postoperatively and a duration of approximately 6 days. On multivariate analysis, POH was significantly associated with POD, and the adjusted odds ratios indicated that patients with POH were more likely to develop delirium (OR, 5.61; *p* = 0.006). Advanced age (OR, 1.11; *p* = 0.002), emergency surgery (OR, 8.31; *p* = 0.001), and on-pump coronary artery bypass grafting were identified as risk factors of POD. Patients who developed delirium were typically older, more likely to be male, and had higher morbidity rates than those who did not.

**Conclusion:**

POH is significantly associated with delirium in critically ill patients after cardiac surgery. Surgical complexity and advanced age contribute to the risk of developing POD and poor postoperative outcomes.

## Background

The postoperative phase is a critical period that can immensely harm patients. Postoperative delirium (POD) is associated with cognitive impairment and other sequelae [1]. POD is a common complication of cardiac surgery that can significantly compromise the quality of postoperative care [2]. The number of major cardiac surgeries among elderly patients has been increasing owing to a higher prevalence of comorbidities, more severe illnesses, and poorer clinical outcomes after cardiac surgery in this aging population. POD is more frequently observed in older patients [3, 4].

Additionally, the high incidence of complications during postoperative critical care not only involves significant financial costs but also reduces the patient’s quality of life after hospitalization [5]. POD represents an acute and fluctuating disturbance of attention with unclear pathophysiology, and currently, no definitive preventive or therapeutic measures are available [6, 7]. POD is a multifactorial condition influenced by factors such as age, preexisting cognitive impairment, and the stress of surgery and has been correlated to temperature management and blood pressure fluctuations [8].

POD is a strong predictor of postoperative cognitive decline development and typically occurs within the first 3 postoperative days, whereas POCD occurs at the end of the first week. POD more significantly affects consciousness and potentially have prolonged impacts on patient health and the healthcare system [9]. Postoperative hypotension (POH) can arise due to various factors such as intraoperative hypotension, blood loss during surgery, changes in fluid balance, race, myocardial infarction, history of percutaneous transluminal coronary angioplasty, and use of angiotensin-converting enzyme inhibitors, angiotensin receptor blockers, or statins [10]. An increased blood pressure fluctuations could predict POD [11, 12]. Major adverse events are associated with POH [13]. Perioperative management of blood pressure influences short- and long-term POD during cardiac and noncardiac surgeries [14]. A mean arterial pressure of < 55 mmHg for a prolonged period is correlated with greater odds of POD [15].

The current study aimed to test the hypothesis that POH contributes to the incidence of delirium in patients undergoing postcardiac surgery.

## Materials and methods

### Patient enrollment

This prospective observational study was approved by Nanjing Medical University First Affiliated Hospital on June 9. 2021 (No:2021-SR-242) and conducted at a single center between September 2021 and April 2022. Informed consent was obtained from each participant before surgery. This study was a part of a previous study that determined the incidence of POD. Chinese-speaking adult patients ≤ 65 years old undergoing cardiac surgery were included. Individuals who did not provide informed consent, died before delirium onset, had intraoperative hypotension, or had a history of cognitive impairment were excluded.

The collected data included the occurrence of delirium, clinical parameters (vital signs and mean arterial pressure [MAP]) at the onset of delirium, treatment strategies, and postoperative characteristics. Notably, 34 patients who underwent off-pump coronary artery bypass grafting (CABG) patients were included in this study. These patients were excluded from a previously published article that focused exclusively on cardiac surgery following the use of a cardiopulmonary bypass machine [16].

### Delirium management

Delirium is a clinical disorder characterized by sudden onset and variable trajectory of changes in mental status, attention, and cognition. This condition is frequently identified in individuals admitted to healthcare facilities, especially those undergoing cardiac surgery, and is associated with diverse predisposing and precipitating factors.

In this study, we employed the Confusion Assessment Method for the Intensive Care Unit (CAM-ICU) to assess delirium following surgery. Doctors and intensive care unit (ICU) nurses closely evaluated patients throughout the day to ensure the diagnosis of delirium. The hospital implemented a standardized protocol for managing delirium, which involved the initiation of postoperative sedation through the administration of propofol for the first 5 days, which was subsequently replaced by midazolam. The management of individuals with delirium included the use of benzodiazepines to treat anxiety, haloperidol for hallucination management, and clonidine to manage agitation.

### Hypotension evaluation

POH is characterized by an abnormal decrease in blood pressure or systemic arterial pressure after surgery. In the ICU, the assessment and recording of blood pressure follows a systematic protocol, which includes continuous monitoring through either a blood pressure cuff or an arterial catheter for any exposure diagnosed by the hospital staff. Beyond numerical blood pressure values, the evaluation considered clinical indicators such as hypotension symptoms, heart rate alterations, and other relevant physiological parameters. Blood pressure readings were aligned with the onset of delirium to ensure synchronized evaluation. The MAP was calculated using the following formula:

$${\text{MAP}}=\frac{(2 \times \text{ diastolic blood pressure}) +\text{ systolic blood pressure}}{3}$$

Hypotension was defined as MAP < 65 mmHg), which was used for any exposure [17, 18].

### Surgery and anesthesia

The study included various cardiac surgeries, including coronary artery bypass grafting (CABG), valve replacements, and combined procedures. Notably, 34 patients underwent off-pump CABG, a significant subgroup for analysis.

For surgeries requiring CPB, management focused on maintaining optimal perfusion and minimizing inflammatory responses. Standard protocols included temperature control, appropriate anticoagulation, and close monitoring of vital signs to ensure adequate organ perfusion throughout the procedure.

As for off-pump CABG procedures, the strategy was to maintain cardiac stability without using CPB. Stabilizing devices and careful handling of the heart were used to maintain blood flow while placing grafts. Hemodynamic stability was closely monitored to prevent significant blood pressure fluctuations.

The anesthesia protocol was standardized for all cardiac surgeries. Induction typically involved propofol, followed by maintenance with volatile anesthetics such as sevoflurane or isoflurane. Fentanyl was used for pain management, and muscle relaxants ensured optimal surgical conditions.

### Statistical analysis

SPSS software (version 26.0; IBM Corporation, Armonk, NY, USA) was used for data management and statistical analysis. We also examined the association between POH and POD. The bivariate analysis involved two-sample t-tests for continuous variables and two-sample proportion tests for group comparisons.

Multivariate analysis was performed to assess the significance of differences observed at the descriptive and univariate levels for all variables, with p-values less than 0.2 and indicated by the odds ratio with 95% confidence interval: sex, age, Hb level, heart failure, atrial fibrillation, peripheral vascular disease, cerebral artery disease, smoking, urgent surgeries, emergent surgeries, elective surgeries, on-pump, hypertension, hypotension, hospital stay, morbidity, home discharge, and transfer to other clinics for rehabilitation and treatment.

## Results

**Fig. 1:**
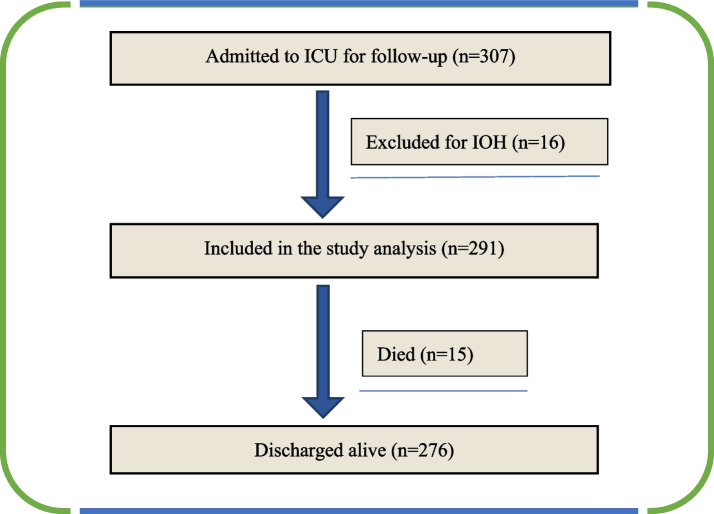
Patient flow diagram. Abbreviations: ICU, intensive care unit; IOH, intraoperative hypotension

This study included 307 patients in total. Of them, 16 patients who experienced intraoperative hypotension or blood pressure fluctuations were excluded from the analysis (Fig. 1). The analysis focused on the remaining 291 participants.

Baseline patient characteristics are summarized in Table 1. The patients who developed POD were notably older and more likely to be male. Oxygen saturation (SO_2_) was 100% in the entire group, except for one case in the delirium group, a 64-year-old female with SO_2_ of 98%. A statistically significant difference in comorbidities and the type and urgency of surgery was noted between groups. Specifically, a significantly higher percentage of patients who developed delirium underwent emergency surgery and on-pump CABG than those who did not. Delirium and hypotension were evaluated at 8-h intervals throughout the day.

**Table 1:** Patient demographics

Variable		Total (291)	No delirium group (230)	Delirium group (61)	*p-*value
Gender	Male	161 (55.3)	123 (53.5)	38 (62.3)	0.002
	Female	130 (44.7)	107 (46.5)	23 (37.7)	
Age (years)		54.09 ± 10.12	52.56 ± 10.47	59.88 ± 5.78	< 0.001
Length of surgery (h)		298.21 ± 41.26	296.73 ± 40.53	303.78 ± 43.81	0.96
BMI (m/kg^2^)		24.53 ± 3.05	24.58 ± 3.06	24.34 ± 3.02	0.78
EF < 50%		96 (33)	74 (32.2)	22 (36.1)	0.28
Hemoglobin (g/dl-1)		12.57 ± 1.95	12.56 ± 2.01	12.59 ± 1.74	0.072
COPD		22 (7.6)	18 (7.8)	4 (6.6)	0.50
Heart failure		27 (9.3)	25 (10.7)	2 (3.3)	< 0.001
Atrial fibrillation		24 (8.2)	15 (6.5)	9 (14.8)	< 0.001
Diabetes		30 (10.3)	24 (10.4)	6 (9.8)	0.78
PVD		32 (11.0)	30 (13.0)	2 (3.3)	< 0.001
Cardiac shock		49 (16.8)	39 (17.0)	10 (16.4)	0.83
CAD		36 (12.4)	24 (10.4)	12 (19.7)	0.02
Smoking		85 (29.2)	64 (27.8)	21 (34.4)	0.06
Urgency of surgery	Urgent	75 (25.8)	64 (27.8)	11 (18.0)	0.121
	Emergent	74 (25.4)	33 (14.3)	41 (67.2)	< 0.001
	Elective	142 (48.8)	133 (57.8)	9 (14.8)	0.001
Type of surgery	On-pump CABG	54 (18.6)	34 (14.8)	20 (32.8)	< 0.001
	Off-pump CABG	34 (11.7)	23 (10.0)	11 (18.0)	0.83
	Other types (Valves & other combined)	203 (69.8)	173 (75.2)	30 (49.2)	< 0.001

*Abbreviations*: *BMI* body mass index, *CABG* coronary artery bypass grafting, *CAD* cerebral artery disease, *COPD* chronic obstructive pulmonary disease, *EF* ejection fraction, *PVD* peripheral vascular disease

Data are given as n (%) and as mean ± SD

### Delirium-related data and postoperative outcomes

Table 2 summarizes the postoperative outcomes and characteristics associated with delirium. Among all patients, 21% (*n* = 61) experienced delirium, with an average onset occurring 4.93 ± 3.40 days after surgery with a duration of 5.94 ± 1.87 days. Morbidities, including cerebrovascular accidents, myocardial infarction, and low cardiac output syndrome, were significantly higher in patients who developed delirium than in those who did not (*p* < 0.001).

**Table 2:** Bivariate analysis of postoperative outcomes

variable		Total (291)	No delirium group (230)	Delirium group (61)	*p-*value
Hypertension		112 (38.5)	85 (37.0)	27 (44.3)	0.10
Hypotension		32 (11)	19 (8.3)	13 (21.3)	< 0.001
MAP		94.6 ± 20.85	94.85 ± 19.56	94.07 ± 25.30	< 0.001
**Delirium-related parameters**					
Incidence		61(21.0)			
Onset (days)		4.93 ± 3.40			
Duration (days)		5.94 ± 1.87			
ICU stay (days)		23.83 ± 6.81	22.83 ± 5.57	25.44 ± 10.01	0.004
Hospital stay (days)		16.34 ± 5.37	15.94 ± 5.18	17.81 ± 5.85	0.25
Morbidity		98 (19.9)	35 (15.2)	23 (37.7)	< 0.001
Mortality		15 (5.2)	11 (4.8)	4 (6.6)	0.27
Discharge	home	203 (69.8)	180 (78.3)	23 (37.7)	< 0.001
	Other clinics	73 (25.1)	39 (17.0)	34 (55.7)	< 0.001

*Abbreviations*: *ICU* intensive care unit, *MAP* mean arterial pressure

Data are given as *n* (%) and as mean + SD

## Indicators of postoperative delirium

**Fig. 2:**
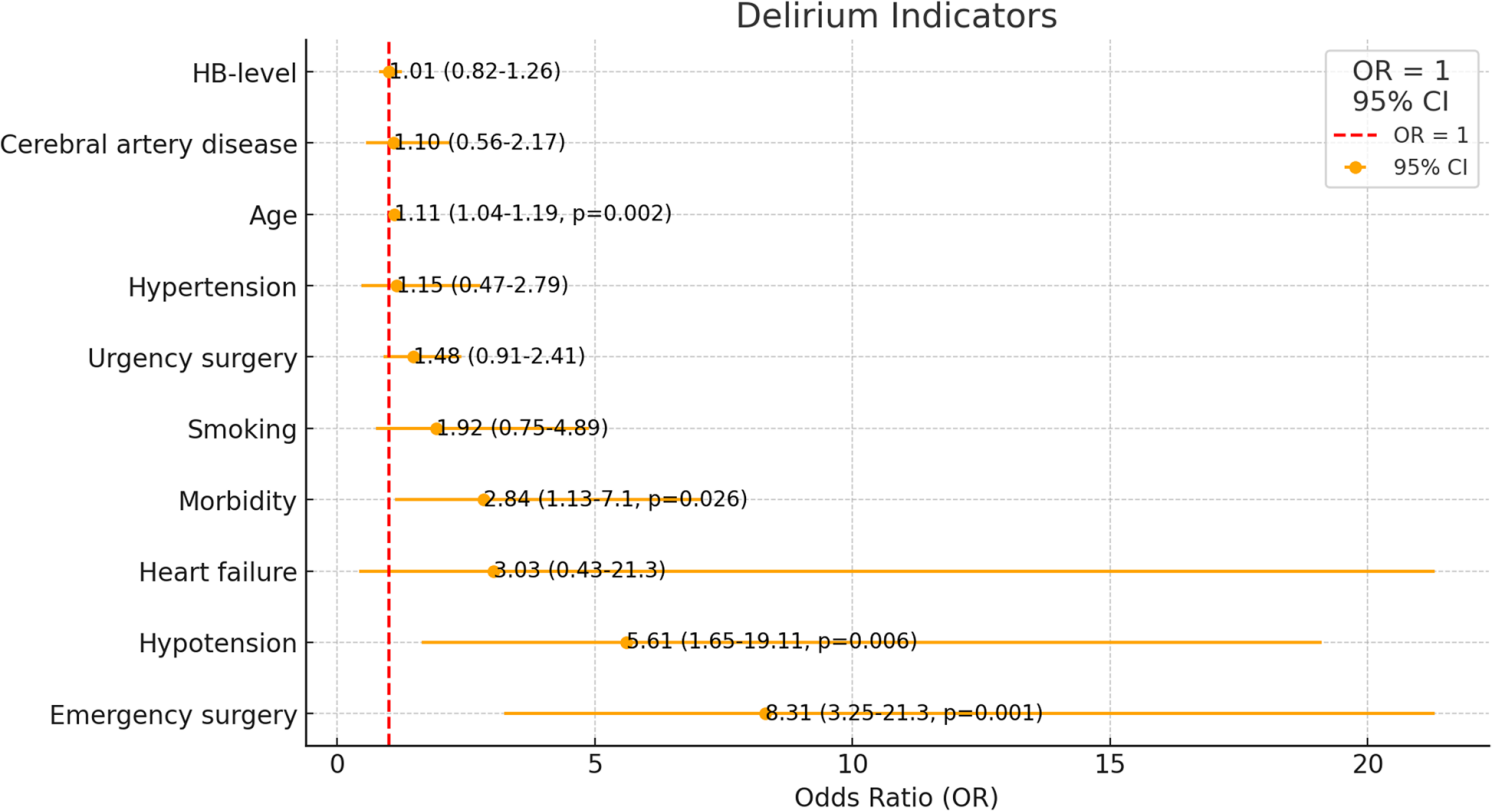
Forest plot showing the odds ratios (OR) and 95% confidence intervals (CI) for various factors associated with postoperative delirium. Significant associations were found with age (OR 1.11, 95% CI 1.04-1.19, *p* = 0.002), morbidity (OR 2.84, 95% CI 1.13-7.1, *p* = 0.026), hypotension (OR 5.61, 95% CI 1.65-19.11, *p* = 0.006), and emergency surgery (OR 8.31, 95% CI 3.25-21.3, *p* = 0.001)

The multivariate analysis of delirium indicators revealed several key associations (Fig. 2). Age was a significant factor, with each additional year increasing the odds of developing delirium by 11% (*p* = 0.002). Emergency surgery dramatically increased the risk of delirium, making patients more than eight times as likely to develop delirium (*p* = 0.001). Hypotension also significantly increased the odds, with patients more than five times more likely to develop delirium (*p* = 0.006). Additionally, high morbidity nearly tripled the risk (*p* = 0.026).

However, some of the factors were not significantly associated with delirium: hemoglobin levels, heart failure, cerebral artery disease, smoking, urgent surgery, and hypertension. These findings suggest that while factors such as age, emergency surgery, hypotension, and morbidity are critical in predicting delirium, other factors such as hemoglobin level, heart failure, and smoking do not have a significant impact.

## Discussion

Research and clinical interest in POD, particularly in cardiac surgery patients, has increased tremendously. Delirium, a disabling condition, is likely to cause prolonged cognitive impairment. The postoperative phase is a critical period for patients during which complications such as POD can significantly affect recovery.

In the context of cardiac surgery, the prevalence of POD poses challenges to the quality of postoperative care, specifically in the aging population, in whom major cardiac surgeries are being increasingly performed. This study aimed to investigate the relationship between POH and delirium in patients who underwent cardiac surgery.

In the current adult patient study, an independent association was observed between POH and early-onset delirium, which aligns with our initial hypothesis and differs from the findings of previous studies. However, no correlation was observed, and no relationship between hypertension and POD [19]. Based on our findings, the adjusted models demonstrated a significant association between delirium and POH. The odds ratios exceeded 5 (*p* < 0.006), indicating that individuals who developed POH were highly prone to POD.

Similar to our previous and other published reports, we found that most patients with delirium were older adults [20–22].

The explanation lies in the physiological processes linked to aging, as advanced-age individuals tend to become frail [22]. Therefore, any disabling condition or comorbidity could affect the individual’s cognition and overall health. We also observed a higher prevalence of delirium in patients undergoing emergency surgery. This observation is supported by a previous report [23]. Our study found that delirium was associated with increased morbidity, consistent with findings from previous research [24]. In this investigation, we observed an increased occurrence of delirium in on-pump CABG and other non-coronary cardiac surgeries compared with off-pump CABG. This finding is consistent with that of a previous study, which independently linked the type of surgery operation to delirium [25].

Intraoperative hypotension or fluctuations in blood pressure independently pose a risk for the development of POH, POD, and various complications [26–28]. Individuals who experienced intraoperative hypotension were excluded from the study. The exclusion of patients with intraoperative hypotension aimed to ensure a focused investigation into the specific impact of hypotension on delirium in the ICU setting, thus contributing to the rigor of our study design. Contrary to earlier findings that indicated a correlation between extended operative time and increased risk of POD, our study did not find an association between the two [29].

Statistical differences between the groups regarding ICU stay duration and postoperative complications were noted. Importantly, these results align with those reported in a previous study [30].

Shirvani et al. reported that delirium occurring in the postoperative period after CABG is associated with electrolyte imbalances and metabolic disturbances. One potential rationale for this is that patients undergoing major cardiac surgery often experience blood loss and oxygen saturation fluctuations during the operation, which may account for the onset of hypotension during the postoperative period [31]. Maintaining a baseline regional cerebral oxygen saturation level > 50% is recommended to decrease the likelihood of POD development [32].

POH and POD are significant complications associated with surgical procedures, particularly in older adults. Several mechanisms link POH with POD. Cerebral hypoperfusion due to reduced blood pressure impairs brain oxygen and nutrient delivery and increases delirium risk, particularly in vulnerable populations [33]. Hypotension also triggers inflammatory responses, releasing cytokines like IL-6 and TNF-α, contributing to delirium. Metabolic disturbances, such as hypoxia and acidosis, disrupt neuronal function, whereas sustained hypotension compromises the blood–brain barrier, allowing toxins to affect the brain. Additionally, hypotension alters the neurotransmitter balance, affects cognitive function, and leads to delirium [34, 35].

In our study, all patients underwent major cardiac surgeries under general anesthesia, and delirious patients were treated postoperatively using a standardized protocol for delirium. The use of dexmedetomidine has been identified as being linked to decreased occurrence of POD in the early days after the procedure [36].

Furthermore, our findings also indicate a substantial influence of delirium on postoperative outcomes. Individuals who experienced delirium during their hospital stay experienced more severe postoperative complications than those who did not, which is similar to that of previous studies [37].

Moreover, our study revealed that patients experiencing delirium were more likely to be transferred to other healthcare facilities for ongoing treatment, in contrast to patients without delirium.

Our study had some limitations. First, the timing, duration, and frequency of blood pressure measurement during the postoperative days were not reported in this investigation; however, they were reported for any exposure. Second, hypotension management was beyond the scope of this investigation. Third, the etiology of delirium in the late postoperative period may differ from that in the early postoperative period, and extending the measurements beyond ICU discharge is unlikely to alter our findings. Fourth, its single-center design might have limited the generalizability of our findings to healthcare settings with different protocols and patient demographics. Considering the sample size of 307 patients, excluding 16 participants may have affected the statistical power. A larger sample size would have enhanced the reliability and precision of this study. The variety of surgeries and the use of on-pump versus off-pump techniques.

Finally, our study reported a standardized delirium management protocol without specific anesthesia and medication administration details, introducing variability in treatment approaches that may influence the observed outcomes.

## Conclusions

Our investigation substantiated the study hypothesis, revealing a robust association between POH and delirium in a single center. This report provides novel insights into POD among critically ill patients after cardiac surgery, as no prior study has explored this association in the absence of intraoperative blood pressure fluctuations.

## Data Availability

Data availability statement. Data will be made available on request.
